# Effect of temperature treatment on fruit quality and immunoregulation of Satsuma (*Citrus unshiu* Marc.) during storage

**DOI:** 10.1002/fsn3.1771

**Published:** 2020-08-30

**Authors:** Wenjun Deng, Jinlong Wu, Yurong Da, Zhaocheng Ma

**Affiliations:** ^1^ Key Laboratory of Horticultural Plant Biology (Ministry of Education) Huazhong Agricultural University Wuhan China; ^2^ Zhengzhou Fruit Research Institute Chinese Academy of Agricultural Sciences Zhengzhou China; ^3^ School of medicine JiangHan University Wuhan China

**Keywords:** adverse reaction, immunoregulation, fruit quality, satsuma, storage temperature

## Abstract

Satsuma (*Citrus unshiu* Marc.) is rich in high levels of nutrients and popular for its unique flavor, but the consumption of satsuma is limited by some adverse reactions in human body. Previous studies have mainly focused on the effects of storage temperature on the postharvest quality of satsumas, and little attention has paid to the effect of postharvest satsumas on human body immunoregulation. The purpose of this study was to explore the differences in fruit quality, and the effect of satsuma fruits stored at different temperatures on human health. Satsumas stored at low temperature (5.8°C, LT) and room temperature (23 ± 2°C, RT) for 60 days were sampled every 10 days to measure the fruit quality. Sixty volunteers were recruited for the oral stimulation experiment of satsumas, and then the effect of satsumas on human health was examined through the immunoregulation of RAW 264.7 macrophages. The results showed that compared with RT treatment, LT treatment could delay the degradation of satsuma fruit quality. Both the results of the volunteer experiment and cell experiment indicated that postharvest temperature treatments could reduce the adverse effects of satsuma fruits on human body. These findings indicated that 10‐day storage at room temperature plus subsequent storage at low temperature was the optimal treatment to maintain fruit quality and functional components of postharvest satsumas. This study provides useful information on satsuma consumption and research work from the perspective of immunoregulation evaluation.

## Graphical abstract

This study provided an insight into the dynamic changes of fruit quality and the effect of satsumas stored at RT and LT on the human body and macrophages. It was demonstrated that the adverse symptoms induced by satsuma could be relieved by proper postharvest storage treatment. And proper temperature treatment can delay the degradation of fruit quality of satsuma.

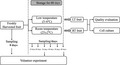

## 1 INTRODUCTION

As the most primarily produced and consumed citrus fruits worldwide, satsuma (*Citrus unshiu* Marc.) fruits are rich in high levels of secondary metabolites. And satsuma is becoming increasingly popular with consumers for their eating quality and health benefits (Sharma, Mahato, & Lee, 2019; Shen et al., 2013; Tietel, Plotto, Fallik, Lewinsohn, & Porat, 2011). After harvest, there is a decline of flavor and sensory acceptability; therefore, satsuma fruit has a short storage life (Tietel et al., 2011). Studies have shown that postharvest treatments can retard aging, delay the deterioration, and extend the shelf life of fruits (Yang et al., 2019). And storage temperature is a crucial factor affecting fruit quality and flavor of satsuma among all the storage treatments (Matsumoto & Ikoma, 2012). Room‐temperature and low‐temperature treatments usually differentially affect the physiological activities of postharvest fruits (Guo, Luo, Han, & Wu, 2019). The optimum storage duration varies with citrus varieties (Cao et al., 2019), and the recommended storage period of satsumas is 2–4 weeks under optimal storage conditions (Obenland, Collin, Mackey, Sievert, & Arpaia, 2011). The minimum safe storage temperature of satsuma is reported to be within 5–8°C, and 20–25°C is usually used to simulate the commercial shelf temperature (Cao et al., 2019; Obenland, Collin, Sievert, & Arpaia, 2013; Tietel, Lewinsohn, Fallik, & Porat, 2012).

Nowadays, health problems are increasingly attracting people's attention, and the nutritional values of fruits have also become the primary focus of researchers and consumers. Deteriorated or degraded fruit will not only affect the fruit taste but also have an adverse effect on human health. Various studies have focused on the impact of storage temperature on postharvest fruit quality, including the basic quality indexes and bioactive components (Obenland et al., 2011, 2013; Rapisarda, Bellomo, & Intelisano, 2001; Rapisarda, Lo Bianco, Pannuzzo, & Timpanaro, 2008). Nevertheless, little research has paid attention to the proinflammatory effects of satsumas during storage. In our daily life, there are often a series of adverse reactions after intaking a certain amount of satsuma, including dry, sore, or hoarse throat, swelling gum, and even oral ulcer, which are similar to the symptoms of inflammation and defined as satsuma‐induced syndrome (SIS) (Ji, Ma, & Deng, 2015). These adverse reactions not only affect people's usual life quality but also limit the consumption of satsumas. The expression of inflammatory mediators could activate macrophages to exert immunostimulatory activity, but overexpression of inflammatory cytokines would induce inflammation. We assume that temperature treatments affect the quality and health‐promoting components of fruit, in turn affecting human health.

Based on this, using satsumas as experiment materials, we combined with volunteer experiments and cell experiments to comprehensively analyze the possible effects of satsumas under different storage temperatures on human health and macrophages. Overall, the objectives of the present study were to evaluate the impacts of two different storage temperatures of satsumas for 60 days on (a) fruit quality, (b) the proinflammatory effect in RAW 264.7 macrophages, (c) human body. Ultimately, this work attempts to determine the optimal storage conditions for maintaining fruit quality with appropriate immune‐stimulating activity and providing available information for growers and consumers to store satsumas.

## 2 MATERIALS AND METHODS

### 2.1 Fruit samples and postharvest treatments

**FIGURE 1:**
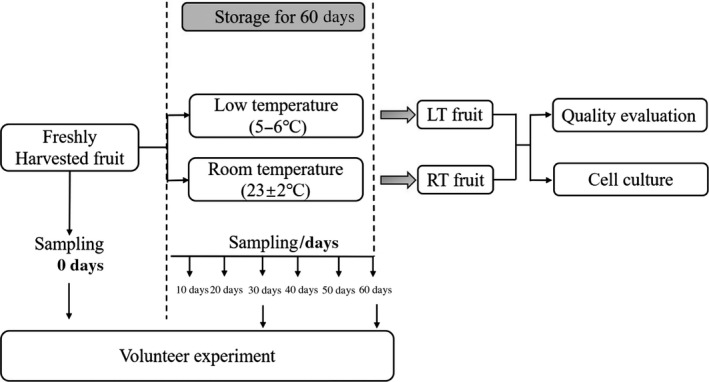
Schematic diagram of the experimental design. The volunteer experiment was conducted every 30 days (before storage, 30 days after storage, and 60 days after storage, respectively). Samples for fruit quality testing and cell experiment were sampled every 10 days (LT, low temperature; RT, room temperature)

Satsuma fruits were harvested from an orchard in Yichang (Hubei, China). After sweating treatment, the fruits without disease and mechanical damage were dipped in the diluted preservative containing imazalil and prochloraz (500 mg/L) for about 1–2 min, cleaned with tap water three times, and air‐dried immediately in a well‐ventilated room. Finally, satsuma fruits were bagged and divided into two groups, which were stored for 10, 20, 30, 40, 50, and 60 days under low temperature (5.8°C, LT) and room‐temperature (23 ± 2°C, RT) conditions, respectively. Fruit sampling was carried out at different storage points. After sampling, the peel and pulp of fruit were quickly frozen in liquid N_2_ and stored at −80°C for further analysis. And the experimental flow chart is shown in Figure 1.

The preparation of satsuma macromolecular water‐soluble substances (WSS) was performed by a step by step strategy as previously described with minor modifications (Yan et al., 2014). After dialysis, macromolecular substances were filtered with a 0.45 μm mixed microporous membrane and then lyophilized.

### 2.2 Measurement of total soluble solids (TSS) and titratable acid (TA)

Fruit juice of satsumas was used to determine the TSS and TA. The TSS content was measured with a digital refractometer PAL‐1 (Atago, Japan) according to the manufacturer's protocol. TSS values were expressed as Brix %. TA was measured following a previous method (Cao et al., 2019). Three repetitions were done for each treatment, with five fruits in each repetition.

### 2.3 Measurement of firmness, weight loss, and color index (CI)

The firmness of the fruit was determined using a texture analyzer. Each fruit was measured evenly along the equatorial plane for three times. The weight loss of satsumas in each treatment was calculated by the difference between the initial and final weights of fruits as follows: weight loss (%) = [(Initial weight‐Final weight)/Initial weight] × 100%. Three repetitions were done for each treatment, with ten fruits in each repetition. Color measurement was performed by a colorimeter at six evenly distributed equatorial sites of each fruit. Color index (CI) was calculated in terms of the formula: CI = 1,000 × a*/(L* × b*). Each treatment contains three repetitions, and each repetition has five fruits.

### 2.4 HPLC analysis of flavonoids

The peel and pulp samples of satsumas were thoroughly ground and lyophilized. The 10 ml 80% methanol was added to 0.1 g peel or pulp powder for ultrasonic extraction for 30 min. After centrifugation, the supernatants were filtered with a 0.22 μm nylon membrane for flavonoid analysis.

Flavonoids were determined by HPLC with an Agilent 1,200 series system (Agilent Technologies) and a C18 column (4.6 × 150 mm, 5 μm) according to Cheng's method (Cheng et al., 2017). As shown in Figure S1, the standards (eriocitrin, narirutin, naringin, hesperidin, neohesperidin, didymin, and nobiletin) (Shanghai yuan ye Bio‐Technology Co., Ltd) were applied for qualitative and quantitative analysis of satsuma flavonoids.

### 2.5 Satsuma oral stimulation challenge in volunteers

sixty healthy volunteers were recruited for the satsuma oral stimulation challenge in an open manner, and they were divided into LT and RT storage groups. Daily experience reveals that excessive consumption of satsuma could cause adverse reactions of the human body, including redness, heat swelling, and pain, and it had a dose‐dependent effect. Therefore, the intake doses for males and females are calculated as the 1.5 kg and 1 kg satsuma fruit divided by average body weight of the volunteer group, and then multiplied by actual body weight, respectively. The volunteer experiment was conducted every 30 days (before storage, 30 days after storage, and 60 days after storage, respectively). For each experiment, volunteers finished the assigned doses at one time on the first day. From day 2 to day 4, volunteers were asked to fill out a standard questionnaire designed in our previous study (Ji et al., 2015). This questionnaire with scoring criteria was used to evaluate the physical symptoms of volunteers, and the symptoms were divided into four levels in the light of the severity (Table S1). It was defined that score ≥ 3 as the adverse reaction of satsumas on the human body. Altogether, the volunteer experiment was carried out once a month for a total of three times. This study has been approved by the ethics committee of Huazhong Agricultural University (Ethical number HZAURA‐2018‐010).

### 2.6 Cell culture and detection of cytokines

The RAW 264.7 macrophages were cultured according to a previously published method (Cheng et al., 2017). Briefly, the RAW 264.7 cells were grown in 25 cm^2^ cell culture flasks in Dulbecco's Modified Eagle's Medium (DMEM) containing 10% fetal bovine serum (FBS) supplemented with penicillin (100 U/ml) and streptomycin (100 μg/ml). The cells were incubated at 37°C and 5% CO_2_‐95% air in a humidified incubator. Near confluent cells were removed with a cell scraper, and then seeded on cell culture plates for further experiments.

To evaluate the immune activity of WSS under different storage conditions, the cells were treated with different WSS samples (0.5 mg/ml). After incubation for 18 hr, total RNA was isolated from the cells with TRIpure reagent (Aidlab, Beijing, China) according to the manufacturer's instructions. And the cDNA was synthesized using a reverse transcription kit (Vazyme). The relative mRNA expression levels of proinflammatory cytokines were detected by using an ABI QuantStudio 6 Flex Real‐time PCR System with SYBR Green PCR Master Mix (Yeasen). Reactions were cycled as follows: 50°C for 2 min, 95°C for 5 min; 95°C for 10 s, 60°C for 30 s (40 cycles). And the primer sequences of proinflammatory factors including cyclooxygenase‐2 (COX‐2), inducible nitric oxide synthase (iNOS), interleukin‐6 (IL‐6), and interleukin‐1β (IL‐1β) are referred to Cheng et al. (2017).

### 2.7 Statistical analysis

All data were expressed as means ± standard error (*SE*). The statistical analysis was performed using SPSS v19 software. The dependent sample *t* test was applied to compare significant differences between the two treatments. Significant differences among different sampling points were calculated using Duncan's test at a 5% level (*p* < .05).

## 3 RESULTS

### 3.1 Effect of storage temperature on the TSS, TA of satsumas

**FIGURE 2:**
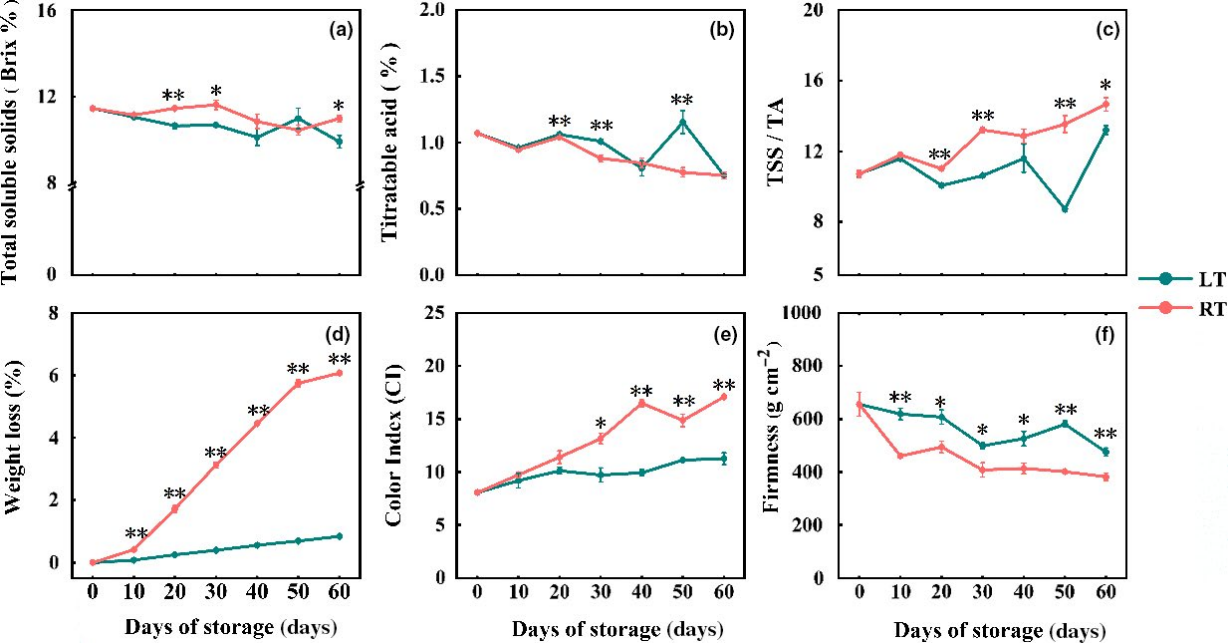
Effect of storage temperature on fruit quality of satsuma. (a) TSS, (b) TA, (c) TSS/TA, (d) Weight loss, (e) Color index, and (f) Firmness. The results were expressed as mean ± *SE*. The statistical significance was applied to compare the differences between two temperature treatments (*p < .05, **p < .01)

The TSS and TA contents of satsuma fruits after storage were determined. TSS contents of fruit stored in RT increased from 11.47% at the beginning to 11.63% after 30 days of storage, and then gradually dropped to 11.0% in the following 30 days (*p* < .05). However, TSS changed differently in LT group, and it reduced from 11.47% to 9.4% (*p* < .01) with the extension of storage time, causing a gradual loss of fruit flavor (Figure 2a).

Before storage, the newly harvested satsuma fruit had TA values of 1.07%, which dropped gradually to 0.75% after 60 days of storage. Still, the TA value of RT (0.88%) was significantly lower than that of LT (1.00%) at 30 days (*p* < .01) (Figure 2b). The decline of TA led to an increase in TSS/TA over the same period. Meanwhile, the values of TSS/TA increased by 36.85% (RT group, 14.67) and 23.23% (LT group, 13.21), respectively, compared with the ratio (10.72) of both groups before storage (*p* < .01) (Figure 2c).

### 3.2 Effect of storage temperature on weight loss, CI, and firmness of satsumas

As shown in Figure 2d, the weight loss rate of satsuma fruits under two storage temperatures went up gradually. The weight loss of fruits in LT group was only 0.84%, while it reached up to 6.07% in RT group after 60 days of storage (*p* < .01), indicating that LT treatment could reduce the weight loss of satsumas. The CI value of fruit peel increased significantly with the storage time from the beginning of 8.06 (Figure 2e). In RT group, it continuously increased to 17.10 (*p* < .01), while the CI value of LT group slowly increased to 11.26. Firmness can be used as an essential indicator to determine the freshness of fruits. And LT treatment could reduce the decrease of fruit firmness compared with RT treatment with the extension of storage time (Figure 2f).

### 3.3 Effect of storage temperature on flavonoid contents of satsuma fruits

**FIGURE 3:**
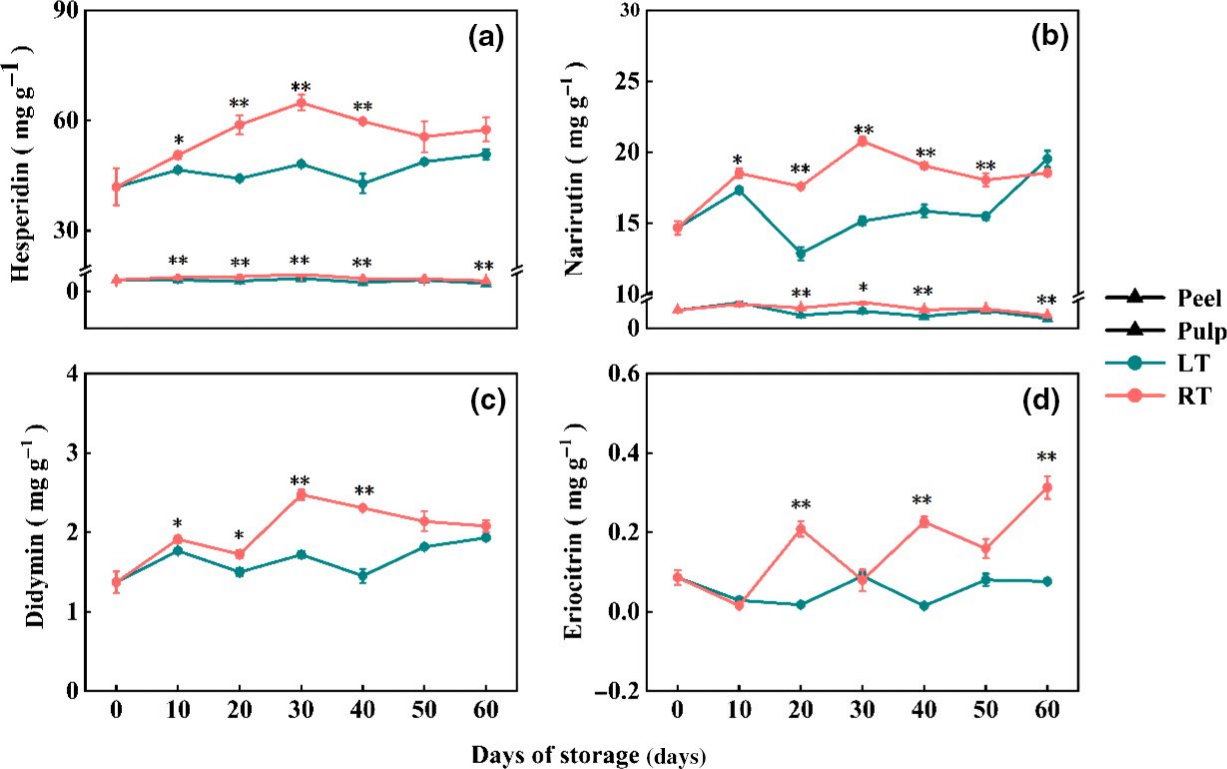
Effect of storage temperature on the flavonoid content of satsuma. (a) Hesperidin, (b) Narirutin, (c) Didymin, and (d) Eriocitrin. The statistical significance was applied to compare the differences between two temperature treatments (*p < .05, **p < .01)

Flavonoids are the main bioactive components widely found in citrus fruit. They contribute to fruit color and taste, and they are related to defense disease and stress resistance. As shown in Figure 3a–d, only four flavonoids were detected in the samples. Among them, hesperidin and narirutin were found in both peel and pulp of satsumas (Figure 3a,b), while didymin and eriocitrin were detected only in the peel (Figure 3c,d). Moreover, the contents of hesperidin, narirutin, and didymin in satsumas stored at RT showed a similar trend of increasing first, reaching the maximum values on 30 days, and then decreasing slowly with the extension of storage time. However, they were still at a higher level than before storage. These three flavonoids in satsuma under LT condition increased continuously during 60 days of storage (Figure 3a–c). Eriocitrin kept stable in LT group, while it increased in a fluctuating manner in RT group (Figure 3d). Besides, the flavonoid level of fruit stored at RT was higher than that at LT.

The total flavonoid of satsuma flesh was also measured. After storage for 60 days, the total flavonoid content under both storage conditions exhibited a slight increase on the 10th day of storage, followed by a decline during the subsequent 50 days of storage (Table 1).

**TABLE 1:** Effect of storage temperature on total flavonoid contents of satsuma pulp

Total flavonoids	Storage duration (days)						
	0	10	20	30	40	50	60
LT	46.78 ± 2.08^ab^	47.73 ± 4.36^ab^	34.40 ± 0.48^cd^	42.50 ± 3.12^abc^	32.02 ± 2.97^d^	48.68 ± 4.15^a^	38.21 ± 1.26^bcd^
RT	46.78 ± 2.08^ab^	48.68 ± 2.65^a^	45.83 ± 5.30^ab^	39.63 ± 3.43^ab^	39.63 ± 1.72^ab^	42.97 ± 0.95^ab^	37.25 ± 2.08^b^

Note

Results are expressed as the mean ± *SE* of three replications. Means with different letter indicate significant differences between different sampling points.

### 3.4 Symptom statistics of oral stimulation experiment of volunteers

**FIGURE 4:**
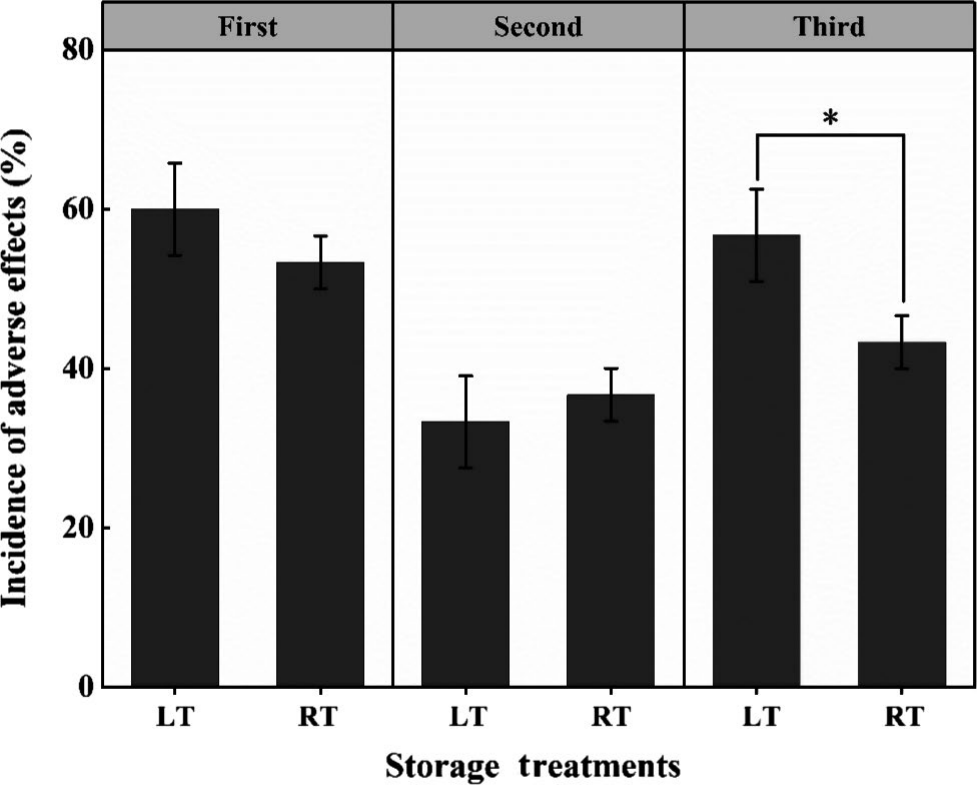
Changes of adverse effects of satsuma stored at different temperatures on volunteers' health. The statistical significance was applied to compare differences in the incidence of adverse effects between two temperature treatments (*p < .05)

**FIGURE 5:**
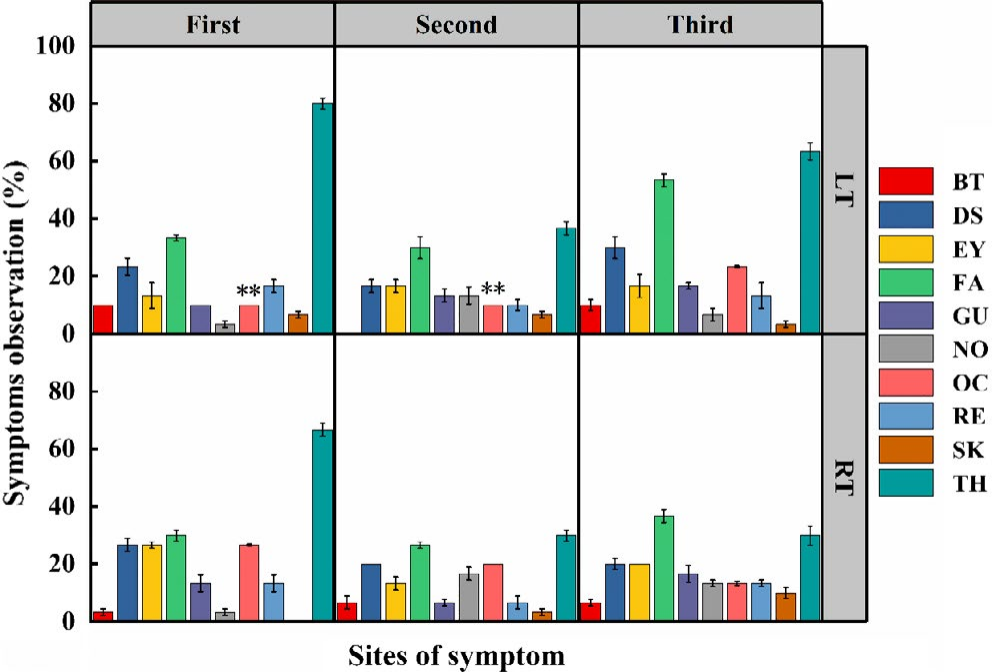
Observation on the health condition of volunteers after the consumption of satsumas. The different color and height of each bar indicate the percentage of symptoms that occur at each site (BT, body temperature; DS, digestive system; EY, eyes; FA, face; GU, gum; NO, nose; OC, oral cavity; RE, respiration; SK, skin; TH, throat). The statistical significance was applied to compare differences in the incidence of adverse effects between two temperature treatments (**p < .01)

The questionnaire results indicated that almost no one showed adverse reactions immediately after finishing the designated doses. In the first volunteer experiment, approximately 56.7% of the 60 volunteers consuming the prestorage satsuma fruits exhibited a range of adverse symptoms after 3 days' observation. Among them, the bad symptom incidence of volunteers was 60% and 53.3% in LT group and RT group (Figure 4). As displayed in Figure 5, these adverse reactions involved various organs of the human body, including the skin, throat, oral cavity, face, eyes, respiratory system, and gastrointestinal system. The throat was the leading site of the occurrence of adverse symptoms of those organs, including dryness, sore throat, or hoarseness, and the incidence of throat symptoms reached 80% (LT) and 66.7% (RT) of the two groups before storage. As one of the pathogenic sites, the incidence of oral cavity showed a significant difference between the two groups. By 30 days, the incidence of adverse reactions of two groups decreased sharply to 33.3% (LT) and 36.7% (RT), respectively, compared with that before storage (*p* < .05) (Figure 4). And the incidence of throat symptoms exhibited the same downward trend, dropping to 36.7% (LT) and 30% (RT, *p* < .05). However, the incidence of oral cavity showed a different trend, which remained stable in LT group, and showed a downward trend in RT group (Figure 5). Interestingly, throat was still the site with the highest bad symptom incidence. On day 60, the adverse symptom incidence in the LT group reaching 56.7% was 23.4% higher than that on day 30 (*p* < .01). However, it only reached 43.3% in RT group, which was 6.6% higher than that on day 30 and was lower than that before storage (Figure 4). The symptom statistics indicated that on day 60, the incidence of throat in LT group reached 63.3%, still higher than the incidence of other organs (Figure 5). However, the incidence of throat (30%) on day 60 in the RT group was lower than that on the face (36.7%), the same as that on day 30. In addition, compared with the increased incidence of oral cavity in LT group, the incidence in RT group showed the opposite decreasing trend.

### 3.5 Effect of storage temperature on immuno‐stimulation of satsuma WSS in RAW 264.7 macrophages

**FIGURE 6:**
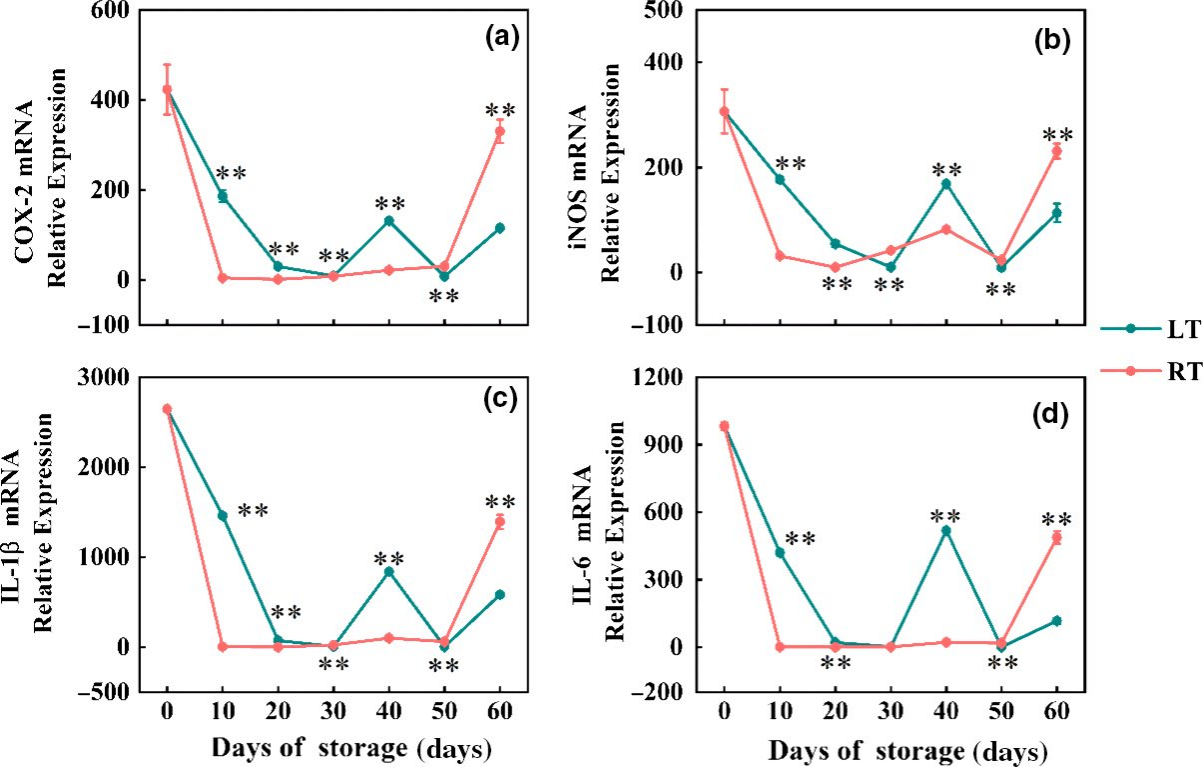
Effect of storage temperature on immune stimulation of WSS in RAW 264.7 macrophages. The cells were treated with WSS (500 μg/mL) for 18 hr, and the expression levels were measured by RT‐PCR. (a) COX‐2 mRNA expression, (b) iNOS mRNA expression, (c) IL‐1β mRNA expression, and (d) IL‐6 mRNA expression. The statistical significance was applied to compare differences in the incidence of adverse effects between two temperature treatments (^*^ *p* < .05, ^**^ *p* < .01)

Our previous studies have shown that the WSS in satsuma fruit could activate macrophages to increase mRNA expression of cytokines and show a proinflammatory effect (Yan et al., 2014). However, excessive immune stimulation can lead to an inflammatory response. As illustrated in Figure 6a–d, the expression levels of cytokines dropped sharply of both groups during 30 days of storage (*p* < .01). However, the RT group exhibited a significantly higher degree of drop than the LT group on the 10th day. From day 30 to day 40, the expression levels of cytokines were stable in the RT group, but they displayed a tendency of increasing first and then decreasing in the LT group. The expression in the RT group exhibited a significantly higher degree of increase than that in the LT group from day 50 to day 60, and both were lower than that before storage (*p* < .01). Our results showed that postharvest temperature treatment could reduce the proinflammatory effect of satsumas.

## 4 DISCUSSION

Satsuma is a typical nonclimacteric citrus fruit with high economic value. Since postharvest fruits will experience a process of senescence and quality deterioration, resulting in rapidly declined sensory acceptability, the shortened satsumas' storage life, especially the decreased flavor quality (Tietel et al., 2011). Previous studies have shown that appropriate postharvest treatment can effectively maintain fruit storage quality and reduce fruit losses (Cao et al., 2019). As one of the most essential factors for postharvest treatment, storage temperature can affect the respiration and other metabolic activities in fruits and vegetables, thus influencing their flavor quality and shelf life (Chaudhary, Yu, Jayaprakasha, & Patil, 2017), which has been confirmed in some studies of various citrus fruits (Rapisarda et al., 2001; Sun, Singh, Tokala, & Heather, 2019; Tietel et al., 2012). The postharvest quality of satsuma fruit is generally indicated by its appearance quality (Fruit size, shape, color, etc.) and internal quality (flavor, TSS, TA, functional components, etc.). Fruit quality could determine its nutritional value, which in turn can have a vital impact on human health. To the best of our knowledge, this is the first study focusing on the effects of postharvest satsumas stored at different temperatures on human immunomodulation.

In general, deterioration caused by fungi and thin skins is the main factor affecting the postharvest quality and consumption of satsumas. Infected fruits are neither suitable for long‐time preservation nor long‐term transportation. Postharvest temperature treatment would affect the decay rate, rind color, and acid contents of Ponkan fruit (Cao et al., 2019). Low‐temperature storage is the most frequently used postharvest technology to maintain the fruit quality and extend storage life because low‐temperature storage can lead to high emission of volatile compounds from fruits, which plays a key role in inducing cellular defense mechanisms against pathogenic infection (Lado, Gurrea, Zacarías, & Rodrigo, 2019). Besides, the application of postharvest preservative measures, such as polyamine and edible coating, could maintain fruit quality by improving its firmness and delaying its deterioration processes (Zahedi, Hosseini, Karimi, & Ebrahimzadeh, 2019). Temperature treatment alone is one of the most economical and environmentally friendly measures for farmers and consumers to maintain the fruit quality of satsumas. In the present study, we found that LT was more conducive to maintaining fruit quality, such as inhibiting weight loss, firmness decrease, TSS, TA, and flavonoid content changes in satsumas. At the same time, satsuma fruits stored at different temperatures also had an immunoregulatory effect on the human body and RAW 264.7 macrophages.

Storage temperature greatly affects the quality of postharvest fruits (Hamedani, Rabiei, Moradi, Ghanbari, & Azimi, 2012; Lee, Zhong, & Chang, 2015). Fruit quality is a complex trait, comprehensively affected by sugars, acids, amino acids, volatile compounds, color, and flavor (Yun et al., 2012). TSS, TA, and the firmness constitute flavor quality of fruit, rind color of the fruit determines the external quality of satsuma. During the early storage of satsuma, TSS content increased as the carbohydrates were hydrolyzed and converted into soluble sugars (Nath, Barman, Chandra, & Baiswar, 2013). Afterward, TSS was constantly consumed and decreased because the respiratory rate exceeded the conversion rate during the subsequent storage period. However, TA was directly consumed through neutralization or respiration without any supplementation, resulting in a continuous decrease in TA content throughout the storage (Liu, Liu, Liu, Liu, & Shang, 2018). In the present study, we found that an increase in TSS and a simultaneous decrease in TA increased TSS/TA, which accompanied by the development of off‐flavors. However, LT treatment decreased fruit metabolism, including acid metabolism, and inhibited the increase of the TSS/TA ratio. A high transpiration rate will cause satsumas stored at RT to rapidly dehydrate and soften, resulting in firmness reduction and decay (Lee et al., 2015; Sun et al., 2019). Our research showed that LT‐treated satsumas exhibited a significantly lower weight loss and higher firmness than RT‐treated fruits, indicating that LT treatment could reduce the water loss in fruits, thereby extending storage time. Since the biosynthesis of carotenoids was highly sensitive to temperature, the temperature was reported to affect the fruit color (Tao, Wang, Xu, & Cheng, 2012). Our results indicated that RT treatment deepened the peel color, whereas LT treatment maintained the stability of peel color.

Citrus fruits are excellent sources of flavonoids, which are the most critical functional component and indicators of nutritional quality. Flavonoids are polyphenols biosynthesized from the phenylpropanoid pathway, playing an important role in modulating the responses of plants and fruits to various abiotic and biotic stresses, and the biosynthesis of flavonoids has been reported to be affected by the storage temperature (Chaudhary et al., 2017; Rapisarda et al., 2008). As the major flavonoids in both peel and pulp of satsuma, hesperidin and narirutin were found to be first increased and then decreased drastically in the present study. Previous studies reported that flavonoids might continue to be synthesized in postharvest fruits and vegetables, and the content would increase during storage; however, senescence and dehydration of satsuma fruit in the late storage period might cause the content of flavonoids to decrease (Shen & Ye, 2012). What is more, high level of hesperidin enabled citrus fruit to possess health‐promoting functions, such as the prevention against inflammation (Wang, Chuang, & Hsu, 2008; Zhao, Wang, Lian, Xiao, & Zheng, 2018), and flavonoids could also provide a defensive barrier for fruit against pathogens and insects (Moreno et al., 2018).

As a widely used inflammation model in vitro, the RAW 264.7 macrophage cell line can release proinflammatory mediators to characterize the severity of inflammation. This study found that satsumas stored at both LT and RT for 30 days significantly reduced the immunostimulatory activity of macrophages. This might be attributed to that storage treatments greatly reduce the adverse effects of satsumas on human health by relieving inflammation caused by excessive expression of inflammatory cytokines. However, in the late period of storage, LT storage could promote the upregulation of cold‐resistant genes and the accumulation of cryoprotective proteins (Carmona, Alquézar, Tárraga, & Peña, 2019), thereby upregulating the expression of proinflammatory cytokines in immune cells. The satsumas stored at different temperatures exhibited the differences in fruit quality, and macrophages might be more sensitive to such fruit quality differences at the molecular level than the human body. Therefore, the human body and macrophages responded differently to satsumas.

SIS is likely to be a multifactorial condition, involving numerous different mechanisms, and it varies from patient to patient. As we know, excessive intake of sugars can cause hyperglycemia and may increase the osmotic pressure of cells, thereby resulting in a series of discomfort symptoms of the throat. The abundant organic acids contained in fruits could also cause oral damage (Bartoshuk, Catalanotto, Hoffman, Logan, & Snyder, 2012) and induce the occurrence of the oral syndrome (Coculescu, Tovaru, & Coculescu, 2014). The previous study has shown that hyperglycemia could activate NF‐κB and make it bind to monocytes (Dickinson, Hancock, Petocz, Ceriello, & Brand Miller, 2008) to produce a large number of inflammatory factors, while a low‐sugar, high‐cereal diet can prevent systemic inflammation in diabetics patients (Qi & Hu, 2007). Satsumas are rich in sugars, which may explain why ingesting satsumas causes adverse reactions in the human body. However, we found that the trend of the adverse effects of satsumas (initial decrease and subsequent increase) on human health and macrophages was contrary to the trend of TSS change (first increase and then decrease) with storage time prolonged. Besides, WSS extracted from satsumas by filtering small‐molecule sugars and acids could enhance the expression of cytokines. These results indicated that sugars and organic acids in satsumas might not be the pivotal factors causing adverse symptoms. Meanwhile, our previous research has reported that the proteins extracted from WSS might be the key substances causing inflammation since these proteins could promote macrophages to produce inflammatory cytokines (Wang, Hu, Yan, Ma, & Deng, 2016; Yan et al., 2014). It was also reported that the immune‐modulatory potential of certain fruits and vegetables was highly related to phenolic substances and carotenoids. These substances might have different effects on immune regulation (Colombo et al., 2019; Lin & Tang, 2007; Terao et al., 2019). However, which components in satsuma during storage affect the human body and immune cells need further study.

## 5 CONCLUSIONS

In postharvest processing of fruit, sweating treatment has been widely used to enhance the disease resistance and condition adaptability of fruits. Our results illustrated that the short‐term storage of satsumas at RT not only contributes to the preservation of fruit quality but also reduces the proinflammatory effect of satsumas on macrophages, enabling satsumas to play an appropriate role in immunity enhancement. From the perspective of the dynamic changes in fruit quality and the impact of satsumas stored at different temperatures on human health, this study suggests that newly harvested satsumas should be stored under RT conditions for 10 days before low‐temperature storage to maintain the quality and nutritional functions of satsuma fruit.

## CONFLICT OF INTEREST

The authors declare that there is no conflict of interest to report in this publication.

## ETHICAL APPROVAL

This study's protocol has been reviewed and approved by the ethics committee of Huazhong Agricultural University (Ethical number HZAURA‐2018‐010), and all volunteers gave their informed consent prior to their inclusion in the study. The study conforms to recognized standards of the Ethical Review Methods for Biomedical Research involving Humans adopted by the National Health and Family Planning Commission of the People's Republic of China.

## Supporting information

Supplementary Material
