# Supplementary material for: Effect of temperature treatment on fruit quality and immunoregulation of Satsuma (Citrus unshiu Marc.) during storage
Source: Food Sci Nutr. 2020 Aug 30;8(10):5443–51. doi: 10.1002/fsn3.1771 (PMC7590330; doi:10.1002/fsn3.1771)
Supplement: Supplementary file 1 — Supplementary Material [file FSN3-8-5443-s001.docx]

Figure S1 Analysis of flavonoid standards by HPLC with detection.

Table S1 Scoring standards of the effect of Satsuma on volunteers’ health.


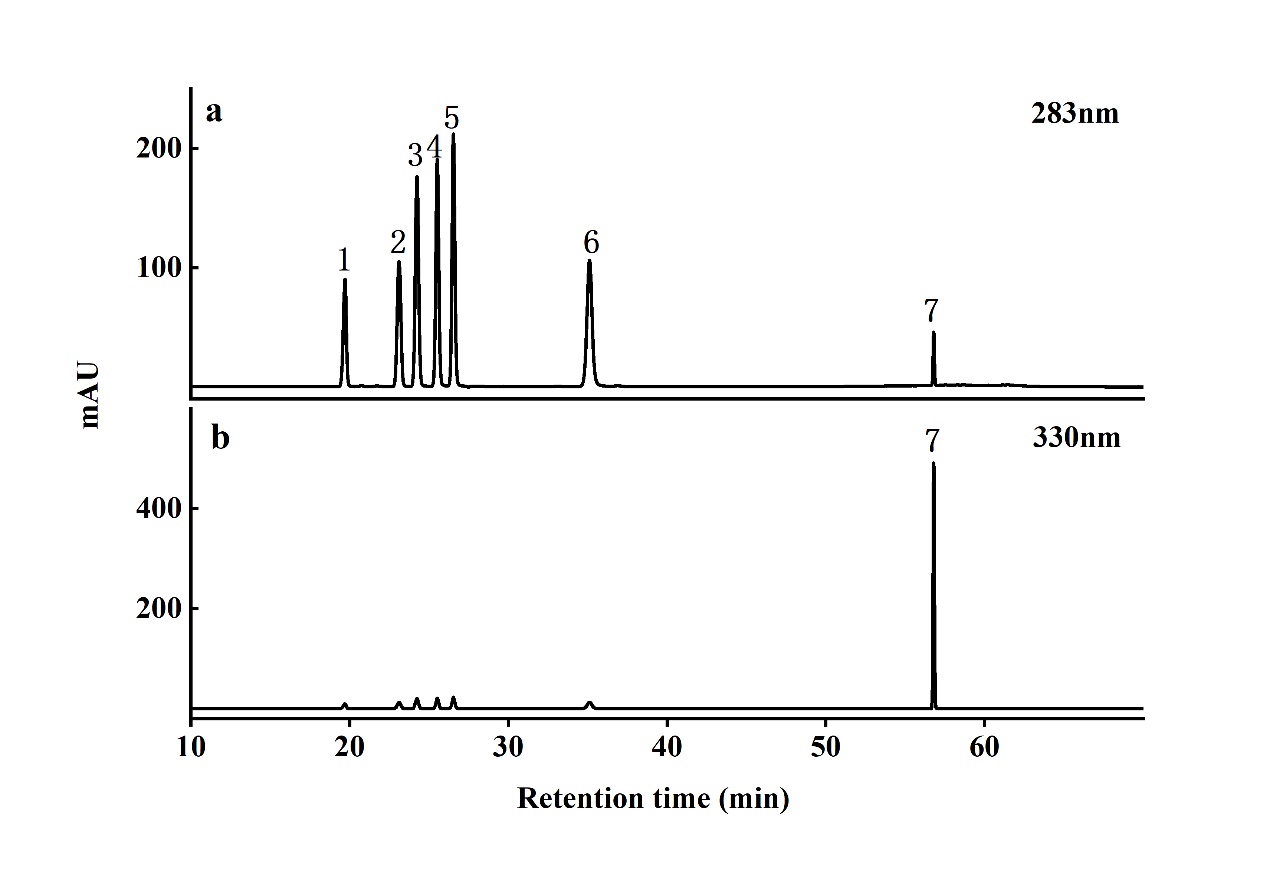


Figure S1 Analysis of flavonoid standards by HPLC with detection at 283nm (a) and 330nm (b). 1: Eriocitrin, 2: Narirutin, 3: Naringin, 4: Hesperidin, 5: Neohsperidin, 6: Didymin, 7: Nobiletin.

Table S1 Scoring standards of the effect of Satsuma on volunteers’ health.

| Severity of symptoms | Score |
| --- | --- |
| Normal | 0-3 |
| Mild | 3-6 |
| Severe | 6-12 |
| Severer | ≥12 |
